# Chitin Nanocomposites for Fused Filament Fabrication: Flexible Materials with Enhanced Interlayer Adhesion

**DOI:** 10.1021/acsami.4c06358

**Published:** 2024-06-28

**Authors:** Alberto Sanz de León, Jose A. Pulido, Natalia Fernández-Delgado, Francisco J. Delgado, Sergio I. Molina

**Affiliations:** Dpto. Ciencia de los Materiales, I. M. y Q. I., IMEYMAT, Facultad de Ciencias, Universidad de Cádiz, Campus Río San Pedro, s/n, 11510 Puerto Real (Cádiz), Spain

**Keywords:** additive manufacturing, fused filament fabrication, chitin nanocrystals, nanocomposites, mechanical properties, interlayer adhesion

## Abstract

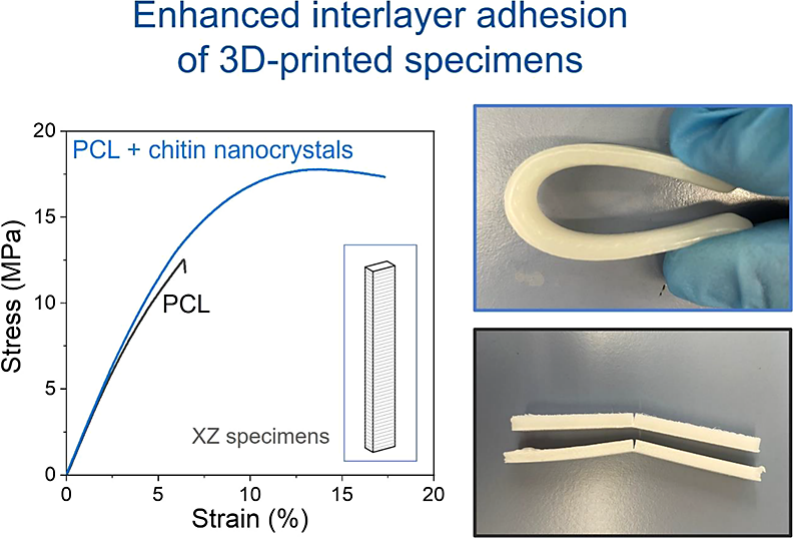

In this work, we present a series of nanocomposites for Fused filament fabrication (FFF) based on polycaprolactone (PCL) and chitin nanocrystals (ChNCs). The ChNCs were synthesized by acid hydrolysis using HCl or lactic acid (LA). The approach using LA, an organic acid, makes the ChNCs synthesis more sustainable and modifies their surface with lactate groups, increasing their compatibility with the PCL matrix. The ChNCs characterization by X-ray diffraction, Fourier transform infrared spectroscopy, scanning electron microscopy, and transmission electron microscopy revealed that both ChNCs presented similar morphologies and crystallinity, while differential scanning calorimetry and thermogravimetric analysis proved that they can bear temperatures up to 210 °C without degrading, which allows their processing in the manufacturing of PCL composites by twin-screw extrusion. Therefore, PCL composites in the form of filaments containing 0.5–1.0 wt % ChNCs were produced and used as feedstock in FFF, and standard tensile and flexural specimens were printed at different temperatures, up to 170 °C, to assess the influence of the ChNCs in the mechanical properties of the material. The tensile testing results showed that the presence of ChNCs enhances the strength and ductility of the PCL matrix, increasing the elongation at break around 20–50%. Moreover, the vertically printed flexural specimens showed a very different bending behavior, such that the pure PCL specimens presented a brittle fracture at 7% strain, while the ChNCs composites were able to bend over themselves. Hence, this work proves that the presence of ChNCs aims to improve the interlayer adhesion of the objects manufactured by FFF due to their good adhesive properties, which is currently a concern for the scientific community and the industrial sector.

## Introduction

Fused filament fabrication (FFF) has become one of the most popular technologies among the different additive manufacturing techniques because it is inexpensive, easy to use, and allows the printing of a wide variety of thermoplastic polymers and composites.^1^ In FFF, a filament is extruded through a nozzle where it is heated and melted before being deposited on the build platform. The objects are generated by adding subsequent layers of the melt material that correspond to a 3D model, previously programmed in a .gcode file. The deposition of each layer is followed by the movement of the platform and the nozzle head, ensuring a good assembly of the next layer.^2^ FFF is currently used in many different industrial sectors because it is a simple, reliable, low-maintenance, and cost-effective process for the production of specific objects and parts with good dimensional accuracy.^3^ For instance, FFF can be combined with medical imaging technologies such as computed tomography or magnetic resonance imaging (MRI) to obtain precise and customized models that can be further printed, being of great interest for biomedical applications.^4^ These designs can be printed using commercially available, biocompatible, and biodegradable materials such as polylactic acid (PLA), polycaprolactone (PCL), or poly(vinyl alcohol) (PVA).^5−7^ This allows developing patient-adapted implants or scaffolds for tissue engineering, reducing waiting times and achieving better performances.^8^

However, one of its current limitations is the anisotropy in the mechanical properties of printed objects due to the poor interlayer adhesion, which can lead to a decrease of more than 50% in tensile strength and stiffness (Young’s modulus) for the same material depending on the direction in which the external load is applied.^9,10^ Classical approaches for improving the mechanical properties of polymers by synthesizing fiber-reinforced composites enhance the intralayer adhesion of printed objects but significantly reduce their interlayer adhesion, often resulting in strength values much lower than those of the polymer matrix.^11,12^ To address this, other alternative strategies have been explored, such as the use of polymer blends with thermoplastic elastomers^13−15^ or star-shaped polymers.^16,17^ These approaches aim to improve interlayer adhesion by increasing the number of supramolecular interactions (van der Waals, hydrogen bonds, or other hydrophobic interactions) between the polymer chains. Other methodologies consist of applying postprocessing after FFF, for instance, thermal annealing^18,19^ or microwave radiation.^20^ Some of these studies also report the improvement of the intralayer adhesion, increasing the overall strength of the material.^15,17,19^ However, a systematic approach that allows obtaining a completely isotropic material by FFF has not been reported to date, limiting the practical applications of the printed objects and parts.

On the other hand, research into more sustainable materials is currently gaining more and more interest due to an increase in the environmental awareness of society and the need of the industry to satisfy this demand. As an alternative to inorganic fillers with a high carbon footprint, chitin is the second most abundant biobased resource on the planet after cellulose.^21^ Chitin consists of a high molecular weight, semicrystalline linear polysaccharide, present in the exoskeletons of many crustaceans.^22,23^ Chitin, and in particular, chitin nanocrystals (ChNCs) are very popular biobased nanomaterials in the biomedical, pharmaceutical, food, and packaging industries due to their high transparency, biodegradability, antioxidant, and antimicrobial properties.^24,25^

ChNCs have been widely used in the synthesis of nanocomposites taking advantage of their good mechanical and functional properties. For instance, Ifuku et al.^26^ developed a series of nanocomposite films based on methacrylic resins with contents of up to 40 wt % ChNCs with enhanced tensile strength and high transparency. Other authors also reported the synthesis of ChNC composites using biodegradable polymer matrixes as polyhydroxy butyrate/valerate (PHBV) or chitosan for food applications.^27,28^ In these studies, the ChNCs increase the mechanical resistance of the films, while they keep the high transparency of the original polymer matrix. ChNCs can also be used as platforms to develop smart materials, such as halochromic films that can act as chemical sensors.^29^ However, these applications are typically based on the manufacturing of films by solvent casting methods, limiting their scalability toward an industrial process.

Alternatively, Salaberria et al. synthesized starch-based^30^ and PLA-based^31^ ChNC biocomposites in a twin-screw extruder, following a more industrial approach. These materials were then used as feedstock in the manufacturing of different specimens by hot compression and injection molding. The starch biocomposites underwent an increase of the mechanical stiffness and strength for ChNCs contents of 5–20 wt %, while an increase in the ductility (elongation at break) of PLA composites loaded with 0.5 wt % ChNCs was observed.

Very recently, a couple of studies addressed the applicability of ChNCs in PLA and PCL-based nanocomposites manufactured by FFF for biomedical applications.^32,33^ In these studies, the authors assessed the influence of the ChNCs in the increase of mechanical properties under compressive stress. However, the well-known adhesive behavior of the ChNCs,^34,35^ and its possible influence on the interlayer adhesion of the polymer matrix was not studied.

In this work, we have developed a series of ChNC nanocomposites using PCL as the polymer matrix. PCL is a ductile and flexible polymer, but it can become brittle when 3D-printed by FFF, due to the poor interlayer adhesion of this process. Hence, the use of ChNCs is proposed to overcome this problem, studying their influence on the interlayer adhesion of different specimens printed. ChNCs were synthesized by acid hydrolysis in two different ways, using HCl or lactic acid (LA). The ChNCs morphology and surface chemistry play a key role in the mechanical properties, which allows the printing of PCL-based objects with high flexibility. This work is the first that reports, to the best of our knowledge, the role of ChNCs as an additive capable of improving the interlayer adhesion in FFF processes, expanding their possibilities of use.

## Materials and Methods

### Materials

Chitin crystalline powder from shrimp shells was supplied by TCI (Japan). Hydrochloric acid (HCl, 37 wt %) and lactic acid (LA, 90 wt %) were supplied by Scharlab (Spain). Polycaprolactone (PCL, *M*_w_ = 60,000 g/mol) pellets were supplied by eSun (China). All the solutions were prepared with Milli-Q water.

### Synthesis of Chitin Nanocrystals (ChNCs)

ChNCs were synthesized via acid hydrolysis following two different methods: (1) A well-established protocol using HCl 3 M at 90 °C for 90 min; (2) a greener alternative using undiluted LA as reaction medium. In this case, the reaction proceeded at 105 °C for 9 h in the presence of a catalytic amount of HCl (0.07 M).^36^ In both cases, a chitin concentration of 33.3 g/L was used. After the indicated times, the reactions were stopped by cooling down with ice. In both cases, the suspensions obtained were centrifuged at 5000 rpm for 15 min and washed with distilled water for several times until the pH of the dispersion increased up to 6–7. The products obtained were freeze-dried to obtain the ChNCs in powder. In this work, the ChNCs obtained by acid hydrolysis with HCl will be labeled as ChNCs-HCl while the ChNCs obtained using LA will be labeled as ChNCs-LA.

### Filament Manufacturing of the ChNCs Composites and 3D-printing by Fused Filament Fabrication (FFF)

**Figure 1:**
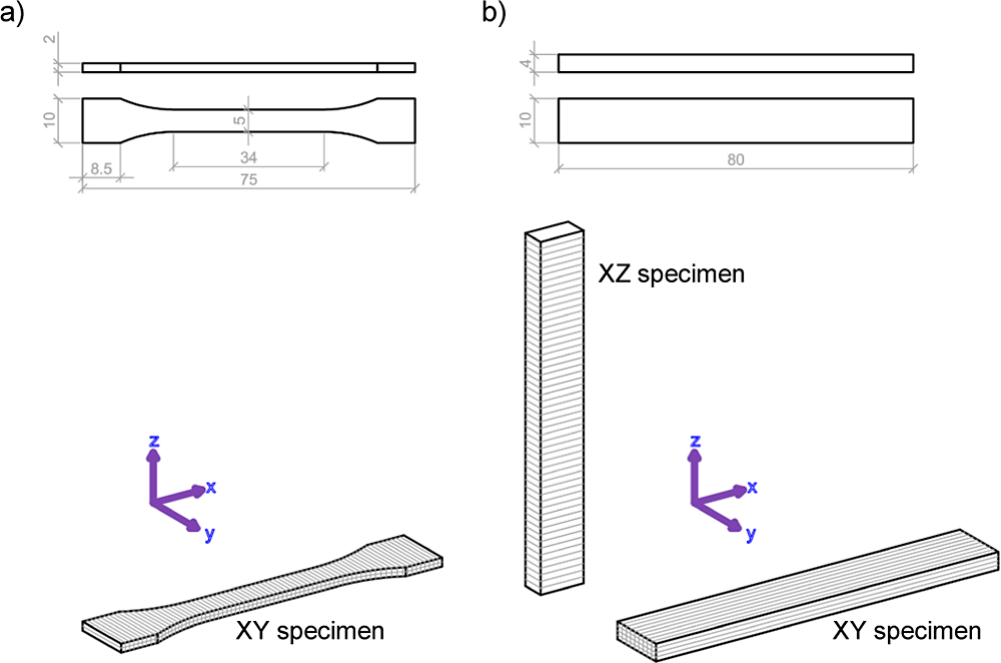
Design and orientation on the building plate of (a) tensile testing specimens and (b) bending specimens. The dimensions are indicated in mm.

PCL filaments for FFF were manufactured in a Scamex Rheoscam D20–20L:D twin screw extruder at 50 rpm with a temperature profile of 80/100/100/100/90 °C, being 80 °C in the dosing area, 90 °C at the end of the extrusion line and 100 °C in the rest of the barrel. The obtained filament was immediately introduced into a water-cooling system and automatically wound into a spool. The PCL, chitin, and ChNCs were previously dried at 60 °C for at least 10 h. PCL composites with 0.5 and 1.0 wt % chitin, ChNCs-HCl, and ChNCs-LA were obtained in the form of filaments with a diameter of 1.75 mm. A filament with only PCL was also manufactured as a control. A minimum amount of 400 g was used in all cases to ensure an adequate compounding of the filaments in the extruder. Then, the filaments were used as feedstock in a Raise 3D Pro-2 FFF printer. Tensile and bending standard specimens according to ISO 527 and ISO 178 were previously designed using a computer-aided design (CAD) software and the .stl file was loaded into the IdeaMaker 4.0.1 software which converts it into a .gcode file that can be recognized by the printer. The printing temperature ranged from 75 to 170 °C and the platform temperature was fixed to 50 °C. All the samples were printed using a nozzle of 0.6 mm diameter, a layer height of 0.2 mm, and an infill density of 100% using a linear pattern. The tensile testing specimens were printed in the XY plane using a raster angle of 90°. The bending testing specimens were printed horizontally and vertically (in the XY and XZ planes, respectively, according to ISO/ASTM 52921) using a raster angle of 0° in both cases. All the XY specimens were printed at 20 mm/s while all the XZ specimens were printed at 10 mm/s. The rest of the printing parameters were kept at their default values. A summary of the different objects printed is presented in Figure 1.

### Characterization of the ChNCs and their Composites

X-ray diffraction (XRD) was measured in a Bruker D8 ADVANCE using a Cu Kα radiation source operated at a voltage of 40 kV with a scanning range of 5–60°. The crystallinity index (CI) of the samples was calculated as follows:^37^
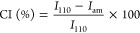
where *I*_110_ is the maximum intensity at 19.1° and *I*_am_ is the amorphous diffraction intensity at 16°. Fourier-transform infrared (FTIR) spectra were recorded in the range of 650–4000 cm^–1^ with a spectral resolution of 4 cm^–1^ in a Bruker Alpha (USA) in transmission mode. Scanning electron microscopy (SEM) measurements were done in a Nova NanoSEM 450. Samples were previously coated with a few nm Au layer in a Balzers SCD 004 sputter coater. The samples for transmission electron microscopy (TEM), in particular, for high-angle annular dark-field scanning transmission electron microscopy (HAADF-STEM) and energy dispersive X-ray (EDX) spectroscopy analysis were prepared by the deposition of a drop containing 1 mg/mL ChNCs-HCl or ChNCs-LA on a holly carbon grid. A TALOS F200X equipped with an EDX microanalysis system (Chemi-STEM) with four SDD detectors was used for the measurements. The accelerating voltage used was 200 kV. The thermal stability of chitin and ChNCs was assessed by thermogravimetric analysis (TGA) in a TA Q50 (TA Instruments, USA). A temperature sweep was performed from room temperature to 600 °C at a rate of 10 °C/min under nitrogen flow. Differential scanning calorimetry (DSC) experiments were carried out in a Q20 (TA Instruments, USA). A first temperature sweep from room temperature to 250 °C at a rate of 10 °C/min, followed by a cooling sweep from 250 °C to room temperature was done. Then, a second heating sweep up to 250 °C at 10 °C/min was done. All the measurements were recorded under nitrogen flow. The mechanical characterization of the printed specimens was done in a Shimadzu AGS-X machine (Germany) using a constant speed of 1 mm/s for both tensile and bending testing, following ISO 527 and ISO 178 standards. The mechanical parameters (i.e., Young’s modulus, elastic limit, tensile strength, elongation at break, flexural modulus, and flexural strength) were obtained for each one of the measured tensile and bending specimens. Young’s modulus was calculated as the slope of the tensile stress–strain curve between 0.05 and 2.5% strain. At least 5 independent samples of each material were tested. Analysis of variance (ANOVA) with a significance level of α = 0.05 and Tukey’s test were performed to determine if there were statistically significant differences between the results.

## Results and Discussion

Synthesis of ChNCs from commercial chitin via acid hydrolysis using concentrated HCl is a well-established approach that can obtain yields of around 60%.^21,25,38^ During this process, amorphous domains of chitin are hydrolyzed and dissolved in the acid medium, while the crystalline domains, insoluble in water, present more resistance to the acid and remain unaltered. Hence, the ChNCs are produced by acid hydrolysis, which eliminates, or at least, highly reduces, the amorphous chitin domains with low molecular order.^38^ Recently, Magnani et al.^36^ presented the synthesis of cellulose nanocrystals from commercial cellulose using lactic acid. Since the molecular and crystalline structures of cellulose and chitin are similar, this approach is expected to lead to similar results for chitin. The use of organic LA reduces the amount of HCl by approximately 40 times, enhancing the sustainability of the synthesis of ChNCs and making it a greener process. The presence of a small amount of HCl ensures the hydrolysis of the glycosidic bonds from the amorphous regions of chitin and catalyzes the esterification of the hydroxyl groups on the surface of the ChNCs with the LA. This allows the formation of lactate groups, decreasing the hydrophilicity of the ChNCs. In this way, this approach also allows the synthesis of ChNCs and their surface modification in a single step. However, since LA is a weaker acid than HCl, harsher conditions are needed (higher temperature and longer time) to hydrolyze the same number of amorphous regions and achieve similar results.

**Figure 2:**
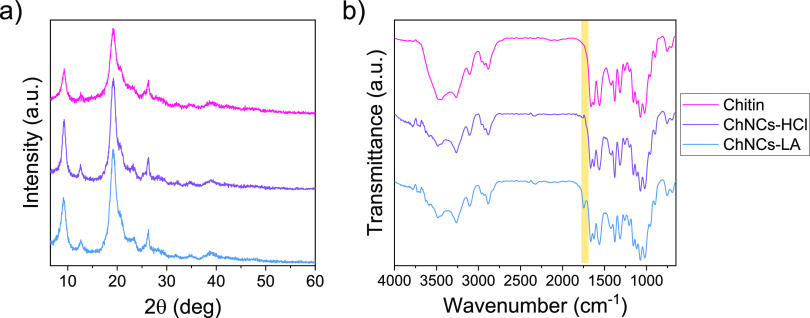
(a) XRD diffractograms and (b) FTIR spectra of chitin, ChNCs-HCl and ChNCs-LA. The peak at 1742 cm^–1^ corresponding to the C=O stretching vibration is highlighted in yellow.

XRD analysis was conducted to see the influence of the different acid hydrolysis treatments on the elimination of the amorphous domains of the commercial chitin. Figure 2a shows the diffractograms of chitin, ChNCs-HCl, and ChNCs-LA, where different characteristic diffraction peaks can be observed at 9.3° (020), 12.6° (021), 19.1° (110), 23.1° (130), and 26.3° (013). These signals remain after both hydrolysis approaches, matching well with those of alfa-chitin,^39^ demonstrating that these strategies allow obtaining the ChNCs without altering their crystalline structure. The CI showed an increase from 73% for commercial chitin to 89% for ChNCs-HCl and 87% for ChNCs-LA, evidencing a significant removal of the amorphous regions that bind the ChNCs into larger structures. CI values obtained for ChNCs are rather similar regardless of the approach used, proving that hydrolysis using LA is virtually as efficient as with HCl.

The chemical composition of the ChNCs was studied by FTIR spectroscopy (Figure 2b). All the materials show similar spectra, including chitin characteristic vibration signals such as O–H stretching at 3439 cm^–1^, N–H stretching at 3259 and 3100 cm^–1^, CH_2_ and CH_3_ stretching at 2885 cm^–1^ and amide I, II, and III stretching at 1654 and 1622, 1553, and 1259 cm^–1^, respectively.^40^ This proves that, as expected, none of the hydrolysis methods altered the chemical structure of the chitin, even though harsh acidic conditions were used. More importantly, in the case of ChNCs-LA, a new peak at 1742 cm^–1^ is observed. This peak corresponds to the C=O stretching vibration of the lactate group, present in the surface of the ChNCs-LA, due to the esterification of the hydroxyl groups with LA, evidencing that the surface modification during the synthesis of ChNCs is achieved, in agreement with previous reports.^36^

**Figure 3:**
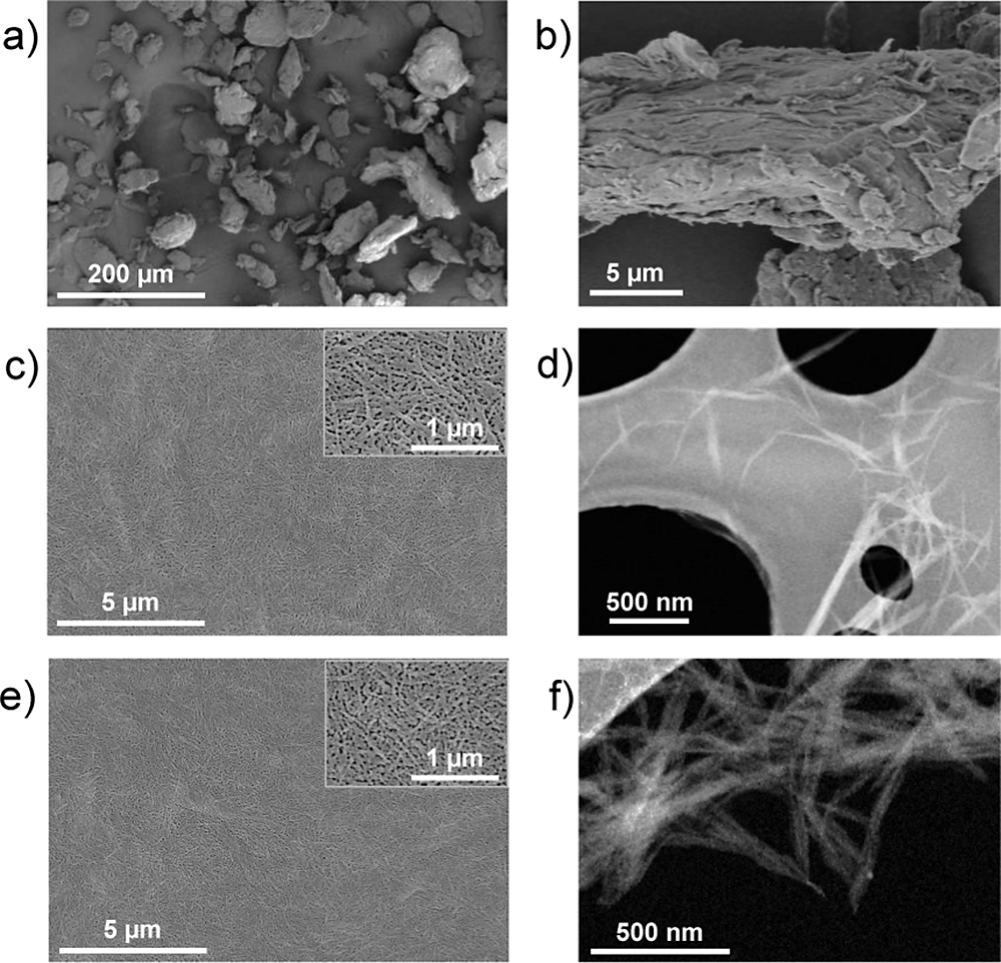
(a) Low and (b) high magnification SEM images of chitin; (c) SEM and (d) HAADF-STEM images of ChNCs-HCl; (e) SEM and (f) HAADF-STEM images of ChNCs-LA.

The XRD and FTIR results were supported by SEM and TEM images of the ChNCs. Figure 3a, b shows the morphology of chitin at different magnifications. Commercial chitin consists of particles with various morphologies and sizes ranging from tens to hundreds of microns. The higher magnification image reveals some fibrillar nanostructures grouped into larger particles that also contain the amorphous domains. Figure 3c,e shows a homogeneous distribution of the ChNCs after acid hydrolysis with either HCl or LA. No significant morphological differences are observed between the two different ChNCs synthesized, proving that the crystalline domains initially present in the commercial chitin have been separated into individual ChNCs, caused by the removal of the amorphous phase, in agreement with our XRD results and with previous reports.^38,41^ Higher resolution HAADF-STEM images in Figure 3d,f show that the ChNCs-HCl and ChNCs-LA possess similar morphologies regardless of the hydrolysis approach followed. The individual ChNCs can be clearly distinguished in both cases, although in the case of ChNCs-LA the individual nanocrystals show a higher tendency to form larger nanofibers or similar nanostructures. This may be key for better performance as the adhesive of reinforcements when embedded in a polymer matrix. Specifically, the ChNCs-HCl possess an average thickness of 27.4 ± 7.5 nm and an average length of 222 ± 69 nm while ChNCs-LA has an average thickness of 24.9 ± 10.2 nm and an average length of 193 ± 65 nm.

**Figure 4:**
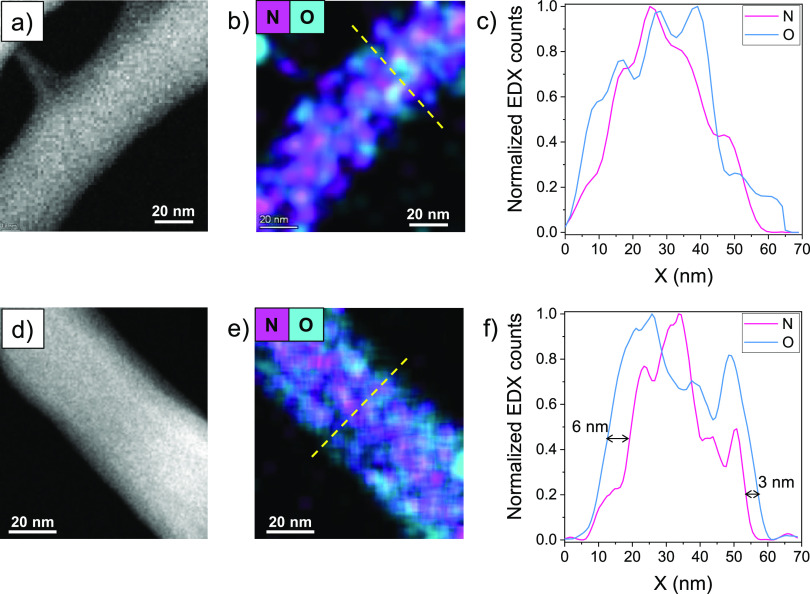
(a) HAADF-STEM image, (b) EDX mapping, and (c) normalized profile EDX counts of one ChNC-HCl; (d) HAADF-STEM image, (e) EDX mapping, and (f) normalized profile EDX counts of one ChNC-LA.

The ChNCs have been further studied by EDX to analyze the surface modification by the lactate groups of the ChNCs-LA. Figure 4a, b shows the HAADF-STEM image and the EDX mapping of N and O of one ChNC-HCl. In this case, both elements, naturally present in the chitin structure, are found homogeneously along the whole nanocrystal studied. The intensity profile analysis in Figure 4c reveals a random distribution of N and O across the nanocrystal, showing that N is also found at the very edge. However, in the EDX mapping of Figure 4e, corresponding to ChNCs-LA, the N is located preferentially at the center of the nanocrystal, while the O signal is also located at the very edge of the crystal. To show this information more clearly, the EDX intensity profiles are presented in Figure 4f. The black arrows in this graph evidence the lack of N EDX counts at the edge of the ChNCs-LA. This can be correlated to the presence of lactate groups, which are composed only of C and O. The analysis of these results indicates that this coating is quite uniform around the ChNCs-LA, with a thickness of approximately 3–6 nm.

**Figure 5:**
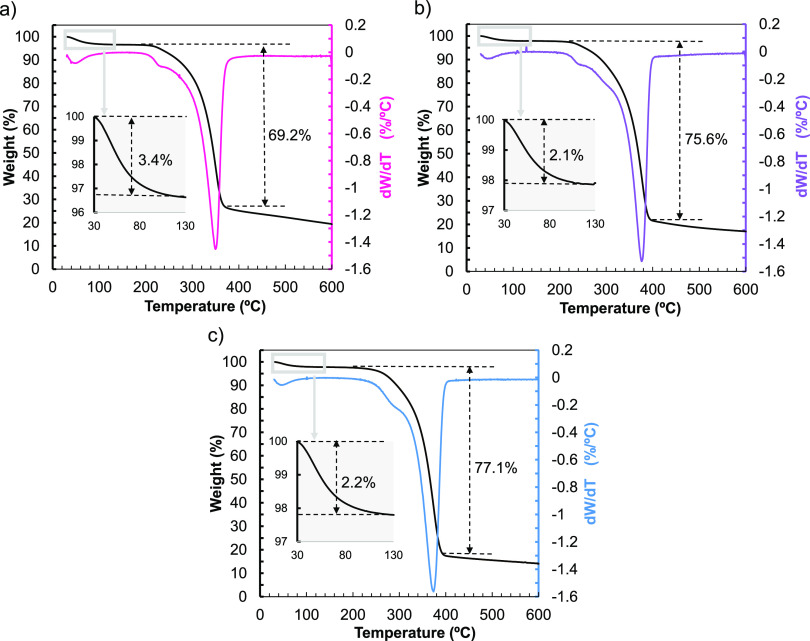
TGA and DTG thermograms of (a) chitin; (b) ChNCs-HCl and (c) ChNCs-LA.

The ChNCs synthesized in this work will be used as additives in the synthesis of nanocomposites by extrusion and subsequent 3D printing by FFF, where the polymer will be melted. Therefore, the thermal characterization of the ChNCs by TGA and DSC is key to determining their degradation temperature and establishing an upper-temperature threshold.^42^ The TGA curves presented in Figure 5 illustrate two independent steps of weight loss for all the materials studied: Initially, a weight loss of 2–3 wt % is observed from room temperature up to 130 °C, associated with the evaporation of water.^43^ This loss is observed in all the samples studied and is slightly higher for the commercial chitin, implying that the ChNCs retain less water, even though these differences are around 1 wt %. No significant differences were observed between ChNCs-HCl and ChNCs-LA in this temperature range. Subsequently, a significantly greater decrease in weight is observed for chitin, ChNCs-HCl, and ChNCs-LA at 200–370 °C, 210–390 °C, and 210–400 °C, respectively, with associated weight losses of 69, 76, and 77 wt %. The weight loss at this stage is attributed to the thermal degradation of the chitin backbone.^44^ The derivative of the thermogravimetric curves (DTG) is also provided, indicating a shift in the temperature of maximum degradation from 350 °C for commercial chitin to 376 and 373 °C for ChNCs-HCl and ChNCs-LA, respectively. This indicates that the thermal stability of the ChNCs is higher than that of the commercial chitin, likely due to the amorphous domains requiring less energy to degrade, as the intermolecular interactions are weaker than in the crystalline regions.

**Figure 6:**
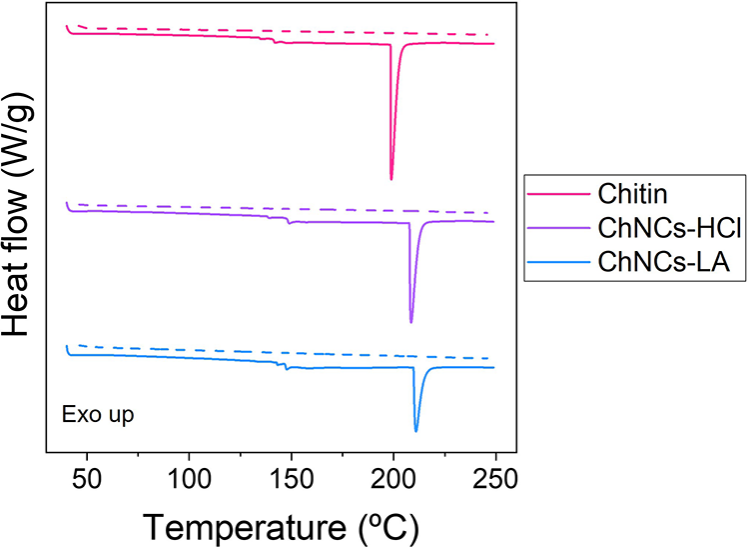
DSC thermograms of chitin, ChNCs-HCl and ChNCs-LA. The solid lines represent the first heating sweep while the dashed lines represent the second heating sweep after cooling down from 250 °C to room temperature.

The TGA analysis is complemented with the DSC results, presented in Figure 6. In this case, the samples were previously dried to avoid the possible influence of water evaporation. A subtle thermal transition at 140 °C can be observed in all the cases. This small variation is similar to that observed for the glass transition temperature (*T*_g_). However, this transition is irreversible, as it is not observed in the second heating sweep, even when the samples were heated slightly above this value in the first heating sweep (e.g., 150–180 °C). Although there is not a strong consensus about this, some authors claim that the *T*_g_ of polysaccharides is above their thermal degradation temperature^45^ and this transition is related to an irreversible, thermal relaxation of the polysaccharide chains without any changes in their chemical structure.^46^ More importantly, all the samples also exhibit an endothermic peak at 199, 209, and 211 °C, respectively with associated enthalpy values of 68.9, 56.8, and 40.5 J/g for chitin, ChNCs-HCl and ChNCs-LA. The temperature is approximately 10 °C lower for chitin than for the ChNCs, as confirmed by the TGA results. This difference suggests that the removal of the amorphous phase in the ChNCs increases their thermal stability. Moreover, these thermal transitions are not observed in the second heating sweep, indicating that they are nonreversible. Some authors have reported that the chitin backbone begins to decompose thermally at these temperatures.^47,48^ However, the enthalpy of thermal decomposition for chitin is reported to be much higher, over 2000 J/g^49^ so this is not likely to happen in this case. Alternatively, other authors suggest that the observed transitions may be caused by the elimination of the −OH groups from the polysaccharide rings, as a small weight loss was also observed in this temperature range by TGA.^45^

Therefore, this nonreversible, endothermic peak sets the maximum temperature that chitin or ChNCs can undergo during their processing by twin-screw extrusion and 3D printing. This limits the thermoplastic polymers that can be used as a matrix in the synthesis of ChNCs nanocomposites since many of the polymers used for FFF (ABS, PETG, TPU, PEEK, PEI···) must be printed at temperatures above 200 °C.^50,51^ However, PCL has a low melting point (60–65 °C) and shows no signs of degradation at temperatures below 250 °C so it can be safely printed at temperatures up to 180 °C.^52,53^ This makes PCL a good candidate as a polymer matrix in the synthesis of ChNC nanocomposites.

Then, the composites were synthesized in the twin-screw extruder using a temperature profile that never exceeded 100 °C, which was high enough to ensure adequate flow of PCL in the equipment. The amount of chitin or ChNCs used ranged from 0.5 to 1.0 wt %. In all cases, homogeneous filaments of 1.75 mm diameter, suitable for FFF, were produced. These filaments were then used as feedstock for FFF, optimizing the printing temperature for each material (see Table S1). While PCL could be properly printed at 75 °C, all the composites clogged at this temperature, particularly those with commercial chitin. To address this, the printing temperature was increased to reduce the viscosity of the melt. It was found that all materials could be successfully printed at 170 °C. Additionally, the ChNCs-LA nanocomposites could also be printed at lower temperatures without clogging, down to 100 °C. This is attributed to the greater compatibility of the ChNCs-LA with the polymer matrix due to the presence of lactate groups, of similar nature to the PCL backbone. A similar effect was observed for other composites for FFF after increasing the surface hydrophobicity to enhance their compatibility with the polymer matrix.^54^

The XY tensile testing specimens were printed with a raster angle of 0°, i.e., perpendicular to the external load applied during the tensile tests. This approach aimed to provide initial insights into the potential effect of the ChNCs on interlayer adhesion, specifically between two contiguous deposited strands within the same layer. Moreover, all tensile testing specimens were printed at the same temperature (170 °C) to ensure that the mechanical properties were directly comparable, eliminating the influence of external variables as the printing temperature.

**Figure 7:**
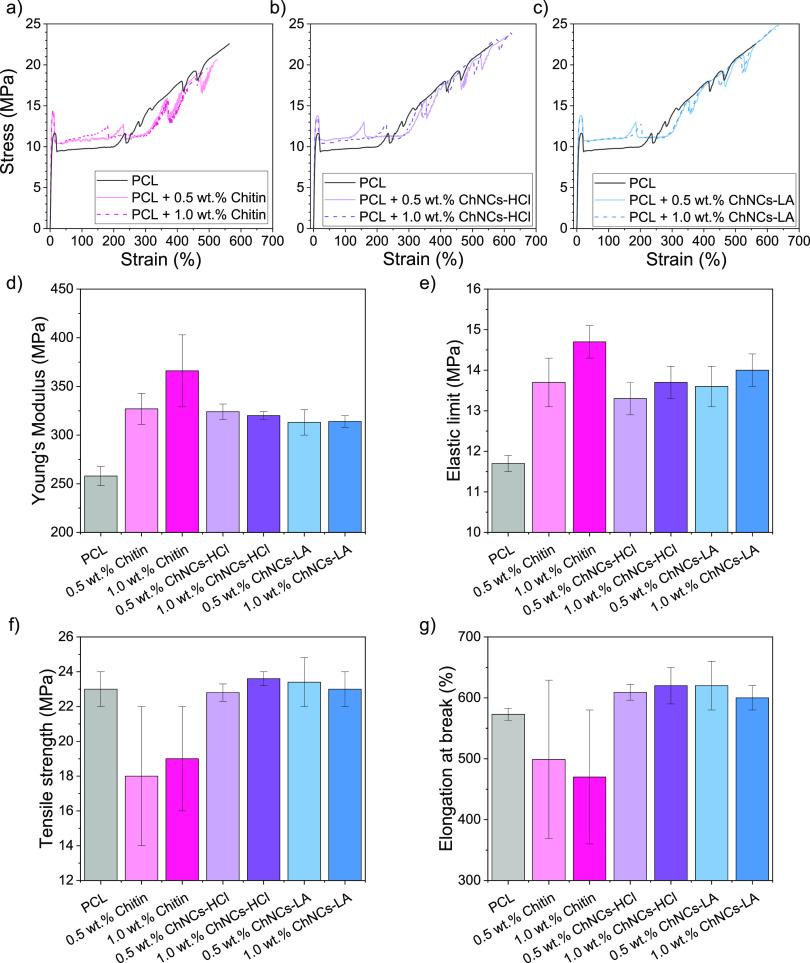
Representative strain–stress curves of tensile tests for (a) chitin; (b) ChNCs-HCl and (c) ChNCs-LA nanocomposites. PCL curve is included in all cases for direct comparison; (d) Young’s modulus, (e) elastic limit, (f) tensile strength and (g) elongation at break values of PCL and PCL nanocomposites dissected from these curves.

The tensile testing results are presented in Figures 7a–c and S1. For clearer interpretation, the curves have been grouped according to the additive used, with PCL always included as a reference. All the tested materials exhibit elastic behavior up to ca. 10% strain, showing a local maximum in stress (elastic limit), followed by a plateau extending to around 200–300%. Subsequently, the stress increases linearly with the strain until the specimen breaks at 450–650%. The materials retain their plastic deformation after breaking, exhibiting the characteristic behavior of a ductile thermoplastic polymer. A series of local maxima are observed in the plastic regime during the tests, which can be related to the raster angle used to print these specimens. This angle, perpendicular to the applied stress, causes localized detachment of the contiguous deposited polymer strands in the same layer, resulting in sudden jumps in the plastic regime of the tensile test. A similar effect was observed by Candal et al.^55^ when performing tearing tests in TPU to evaluate the fracture strength of printed specimens.

The mechanical properties (Young’s modulus, elastic limit, tensile strength, and elongation at break) of PCL and its composites were dissected from the stress–strain curves and are presented in Figure 7d–f. Young’s modulus increases in all cases, indicating that the presence of either chitin or ChNCs enhances the stiffness of PCL. Chitin composites exhibit higher Young’s modulus values than the other composites, and a similar effect is observed for the elastic limit values. All composites have a better elastic limit than the PCL, which is proportional to the filler amount, increasing from 0.5 to 1.0 wt % for all the studied composites. The ChNCs-LA nanocomposites show slightly higher elastic limit values than the ChNCs-HCl nanocomposites, likely due to the surface modification of the ChNCs-LA, which enhances their compatibility with the PCL matrix. A similar effect is observed for the plateau at the beginning of the plastic deformation, which increases by around 3 MPa compared to PCL for all studied composites. Statistical analysis using ANOVA and Tukey’s test confirms that both Young’s modulus and elastic limit values of PCL are significantly different from those of any tested nanocomposites.

However, the tensile strength and elongation at break values notably decrease for the chitin composites, indicating that they act as a noncompatible reinforcement. While chitin enhances the mechanical properties in the elastic regime, it also makes the material much more brittle than the original polymer matrix. This is likely due to the larger size of chitin particles, which limits the mobility of the polymer chains, increasing the stiffness (Young’s modulus) and elastic limit but also facilitating the nucleation of fracture points, leading to material failure. A similar effect has been observed in other nanocomposites, where the presence of chitin increases the stiffness and strength of the material but significantly decreases its elongation, often preventing any plastic deformation,^56^ as seen in other fiber-reinforced composites.^57^

ChNCs-HCl and ChNCs-LA nanocomposites, however, show an increase of ca. 20–50% strain in the average elongation at break value with respect to PCL, although ANOVA and Tukey’s tests indicated that these differences are not statistically different. The tensile strength values do not significantly differ from those of PCL either, suggesting that their reinforcement role might be more effective in the elastic regime. However, the significant differences between the ChNCs nanocomposites and the chitin composites demonstrate that the morphology of the chitin (i.e., nanocrystals vs micron-sized particles) is critical for developing more ductile composites. Furthermore, the differences in mechanical properties between the ChNCs-HCl and ChNCs-LA nanocomposites are not statistically significant, indicating that the main reinforcement effect comes from their nanocrystal shape. Increasing the concentration from 0.5 to 1.0 wt % did not result in significant enhancement in either the tensile strength or the elongation at break for any of these nanocomposites.

**Figure 8:**
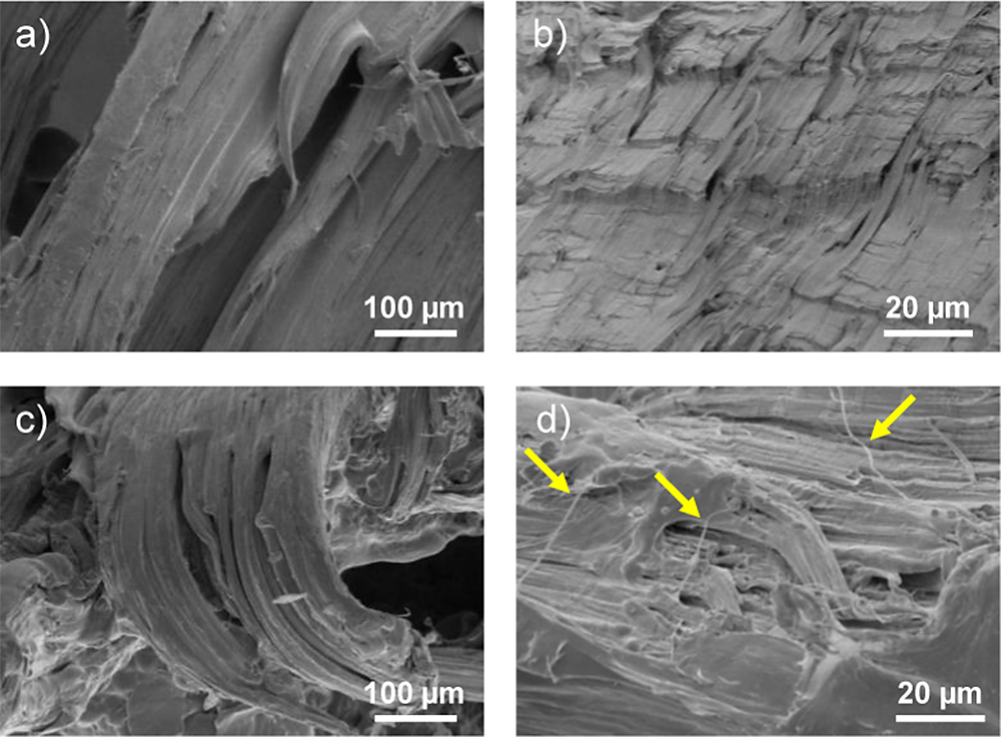
SEM images of the fracture surface of tensile testing specimens of (a, b) PCL and (c, d) 0.5 wt % ChNCs-LA nanocomposites. Yellow arrows in (d) indicate the presence of nanofibers, probably formed by the presence of ChNCs-LA.

SEM analyses of PCL and 0.5 wt % ChNCs-LA nanocomposites were conducted to support the observed mechanical properties. Both materials exhibit a highly deformed surface a low magnifications (Figure 8a,c) because they break at high strain values, above 500%. However, Figure 8d shows that the surface of the ChNCs-LA nanocomposites has some nanofibers likely due to the presence of ChNCs, which may contribute to an increase in the ductility of the material. These fibers are not observed on the surface of pure PCL in Figure 8b.

Salaberria et al.^31^ also observed a similar increase in the elongation at break for PLA nanocomposite films loaded with 0.5 wt % acetylated ChNCs, prepared by hot compression. However, this is the first evidence that the presence of ChNCs improves the ductility of the polymer matrix in FFF, likely due to the hydrogen bonding and other van der Waals supramolecular interactions between the surface of the ChNCs and the backbone of the PCL chains, which can enhance the interlayer adhesion of printed objects.^15^

Considering the flexible behavior of PCL, a more detailed evaluation of the interlayer adhesion was carried out on the nanocomposites studying their mechanical behavior under bending stress. Based on the tensile testing results, 0.5 wt % was established as the optimal concentration of ChNCs, and the bending studies were performed on these composites. All the XY bending specimens were printed at 170 °C. Additionally, PCL and ChNCs-LA nanocomposite specimens were also printed at 75 and 100 °C, respectively, to evaluate the influence of the printing temperature on these two materials that can be printed at lower temperatures (see Table S1 for more details).

**Figure 9:**
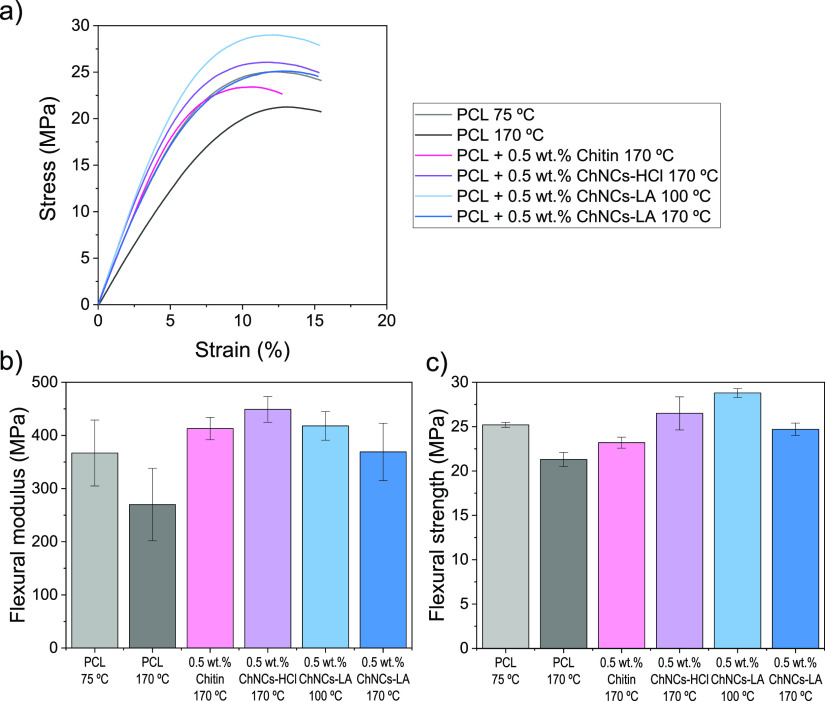
(a) Representative strain–stress curves of flexural tests of XY specimens of PCL, 0.5 wt % chitin, 0.5 wt % ChNCs-HCl and 0.5 wt % ChNCs-LA composites manufactured at different printing temperatures; (b) flexural modulus and (c) flexural stress values dissected from these curves. The printing temperature is indicated in the *X* axis.

Figure 9 shows the bending behavior of the XY-printed specimens, all of which exhibit the characteristic behavior of flexible materials with a maximum stress value of around 10% strain. The flexural modulus and strength of all the nanocomposites tested are higher than those of PCL when they are printed at 170 °C. However, when PCL is printed at 75 °C, its flexural modulus and strength increase significantly, reaching values similar to some of these composites. A similar effect is observed when the ChNCs-LA nanocomposites are printed at 100 °C, indicating that a decrease in the printing temperature enhances the mechanical properties of these materials under bending stress. We hypothesize that the viscosity of PCL (and ChNCs-LA nanocomposites) at 170 °C is probably too low, resulting in poorer mechanical properties at this temperature, even though they can be printed successfully. For this reason, the XZ-printed bending specimens were printed only at the lowest possible temperature for each material.

**Figure 10:**
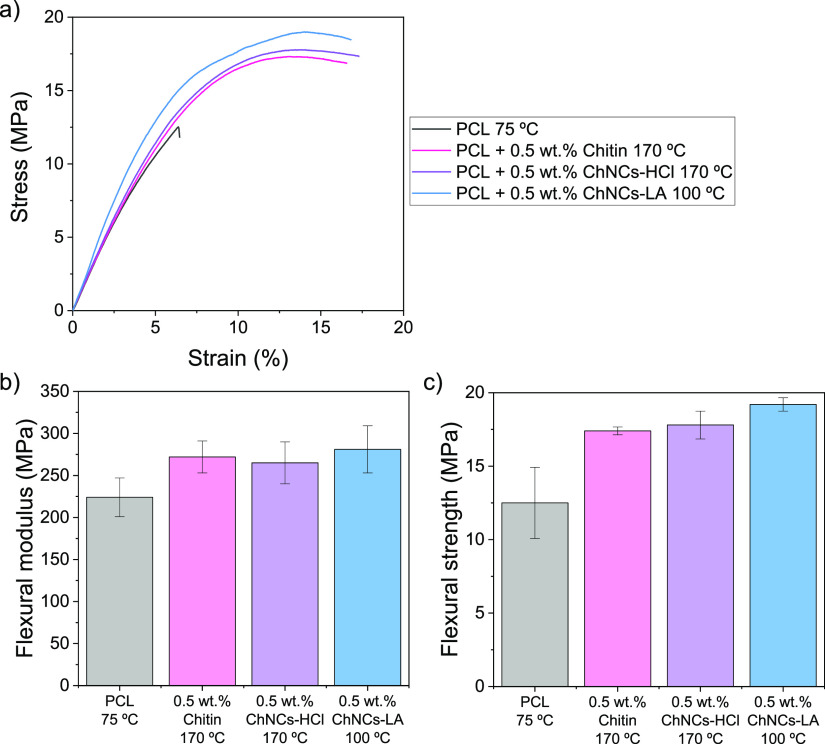
(a) Representative strain–stress curves of flexural tests of XZ specimens of PCL, 0.5 wt % chitin, 0.5 wt % ChNCs-HCl and 0.5 wt % ChNCs-LA composites; (b) flexural modulus and (c) flexural stress values dissected from these curves. The printing temperature is indicated in the *X* axis.

The results from the bending tests of the XZ-printed specimens are presented in Figure 10. All the PCL specimens broke with a brittle fracture at strain values below 10%, with an average elongation at break of 6.9 ± 2.4%. This is an expected behavior for XZ-printed materials by FFF, which typically exhibit weaker behavior when tested in this direction due to the poor interlayer adhesion, a phenomenon widely for printed materials under tensile loads.^15,16,19,58^ However, this is, to our knowledge, the first time that interlayer adhesion has been studied under bending stress. None of the 0.5 wt % composites broke during the bending experiments. The tests were stopped at ca. 15% strain because the specimens started to slide off the support points. ANOVA tests show that the flexural modulus and flexural strength of all the composites studied are statistically higher than those of PCL, as they can withstand larger deformations. The ChNCs improve the interlayer adhesion, achieving better ductility than pure PCL when printed in the XZ direction. ChNCs-LA composites exhibit the highest bending strength values, demonstrating that both the nanocrystal morphology and surface chemistry enhance the compatibility of the filler with the PCL matrix, contributing to increase the interlayer adhesion under bending loads.

**Figure 11:**
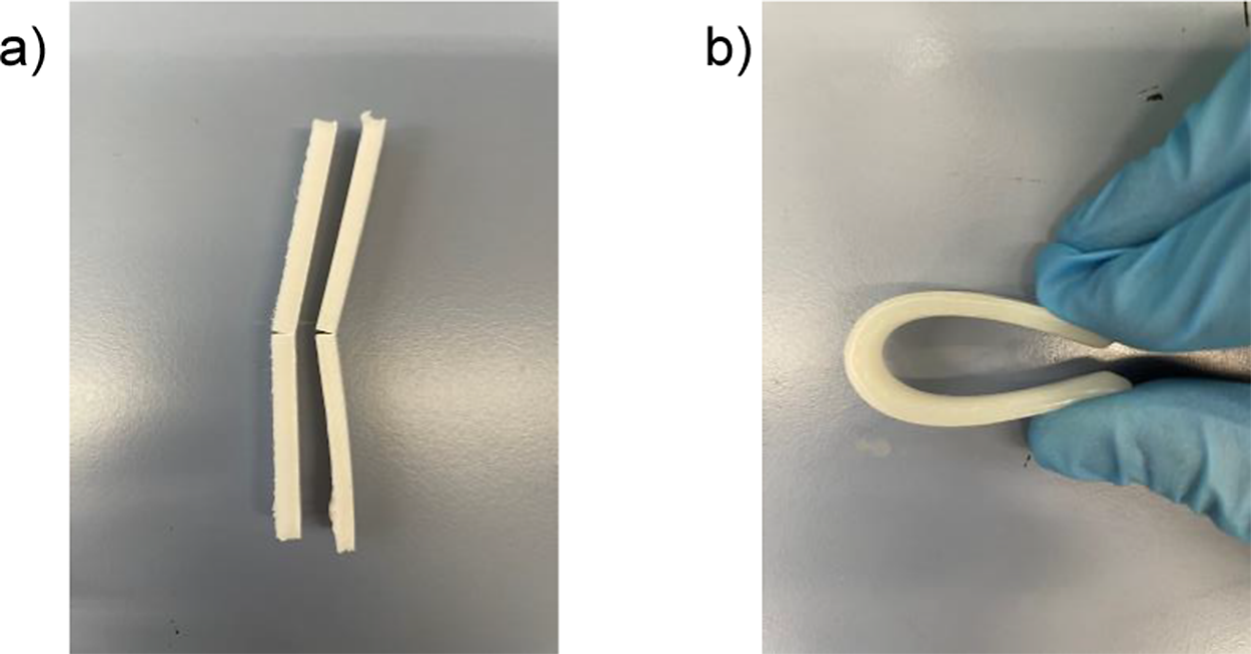
Digital photographs showing the bending behavior of (a) PCL and (b) 0.5 wt % ChNCs-LA nanocomposite. PCL breaks with an abrupt crack for XZ specimens, while the 0.5 wt % ChNCs-LA nanocomposite bears very high strains without breaking.

A digital picture of two PCL XZ bending specimens after testing is presented in Figure 11a, showing a brittle fracture in the center of the sample. Figure 11b, on the other hand, shows a ChNCs-LA specimen that is manually bent so that the two ends of the sample touch each other, demonstrating how the presence of ChNCs increases the interlayer adhesion of objects printed by FFF. This leads to a high improvement in the flexibility of these nanocomposites, expanding their potential use in applications where greater mechanical resistance is required.

## Conclusions

We developed a series of nanocomposites based on ChNCs that improve the interlayer adhesion when printed by FFF, which implies a substantial improvement in the mechanical properties of objects printed with this technology. We demonstrated that the ChNC nanocomposites exhibited superior mechanical properties under both tensile and bending stress compared to those prepared with micron-sized chitin. Furthermore, the ChNCs-LA nanocomposites showed higher compatibility with the PCL matrix due to the similar chemistry of the lactate moieties on their surface. This allowed for the printing of highly flexible materials, eliminating the poor interlayer adhesion observed in the PCL specimens under bending stress.

We believe that these composites have significant industrial potential. Chitin is a highly abundant natural resource, often obtainable as waste from other industries. Our composites demonstrated a substantial increase in the interlayer adhesion of PCL with only 0.5 wt % ChNCs, paving the way for new materials with improved mechanical performance for FFF. Additionally, we show that ChNCs can be processed using twin-screw extrusion instead of solvent casting, enhancing the scalability of these nanocomposites. Therefore, this work broadens the application of ChNCs by demonstrating their effectiveness as a reinforcement to enhance the mechanical properties of objects manufactured via FFF, owing to their adhesive properties. Additionally, ChNCs-LA are biobased nanomaterials synthesized using a green chemistry approach with an organic acid, making them a promising alternative to fossil fuel-derived fillers. Given the biocompatible and biodegradable nature of both ChNCs and PCL, we anticipate that these nanocomposites will significantly impact the biomedical sector. PCL is already widely used in this field, and these nanocomposites can contribute to the development of new 3D-printed, personalized devices with greater mechanical strength.

## Supporting Information Available

Processing parameters tested for each material in the FGF printer and detail of the elastic region of the tensile testing curves (PDF)
