# Supplementary material for: Chitin Nanocomposites for Fused Filament Fabrication: Flexible Materials with Enhanced Interlayer Adhesion
Source: ACS Appl Mater Interfaces. 2024 Jun 28;16(27):35554–65. doi: 10.1021/acsami.4c06358 (PMC11247426; doi:10.1021/acsami.4c06358)
Supplement: Supplementary file 1 — am4c06358_si_001.pdf [file am4c06358_si_001.pdf]

SUPPORTING INFORMATION

Chitin Nanocomposites for Fused Filament Fabrication: Flexible Materials with Enhanced Interlayer Adhesion

Alberto Sanz de León*, Jose A. Pulido†, Natalia Fernández-Delgado, Francisco J. Delgado, Sergio I. Molina

Dpto. Ciencia de los Materiales, I. M. y Q. I., IMEYMAT, Facultad de Ciencias, Universidad de Cádiz, Campus Río San Pedro, s/n, 11510 Puerto Real (Cádiz), Spain.

*Corresponding author: alberto.sanzdeleon@uca.es

†Present address: Grupo de Energía y Química Sostenible (EQS), Instituto de Catálisis y Petroleoquímica (ICP-CSIC), C/Marie Curie, 2, 28049, Madrid, Spain.

Table S1. Different processing temperatures tested for each material in the FGF printer.

Material	Printing temperature (ºC)	Remarks
PCL	75	Printed without problems, uniform printed bead.
PCL	170	Printed without problems, uniform printed bead.
PCL + 1.0 wt.% chitin	120	Clogging.
PCL + 1.0 wt.% chitin	130	Printed with defects. Insufficient flow.
PCL + 1.0 wt.% chitin	150	Printed with defects. Insufficient flow.
PCL + 1.0 wt.% chitin	170	Printed without problems, uniform printed bead.
PCL + 0.5 wt.% chitin	170	Printed without problems, uniform printed bead.
PCL + 0.5 wt.% chitin	120	Clogging.
PCL + 1.0 wt.% ChNCs-HCl	170	Printed without problems, uniform printed bead.
PCL + 0.5 wt.% ChNCs-HCl	170	Printed without problems, uniform printed bead.
PCL + 0.5 wt.% ChNCs-HCl	120	Clogging.
PCL + 1.0 wt.% ChNCs-LA	170	Printed without problems, uniform printed bead.
PCL + 0.5 wt.% ChNCs-LA	170	Printed without problems, uniform printed bead.
PCL + 0.5 wt.% ChNCs-LA	100	Printed without problems, uniform printed bead.
PCL + 0.5 wt.% ChNCs-LA	75	Clogging.

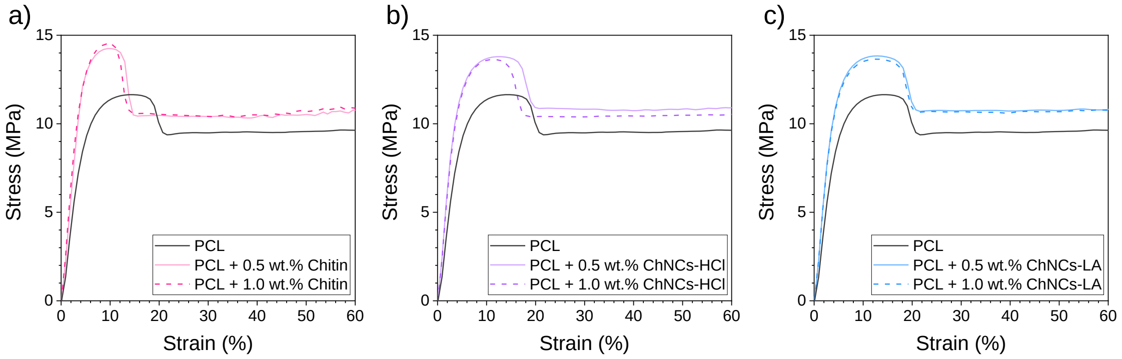

Figure S1. Detail of the stress-strain curves of tensile tests at low strains for a) chitin; b) ChNCs-HCl and c) ChNCs-LA nanocomposites, providing a clearer comparison of their elastic regimes.
